# Positive Role of Delta Neutrophil Index (DNI) as a Prodiagnostic Marker in Cecal Ligation and Puncture (CLP)-Induced Sepsis Murine Model

**DOI:** 10.3390/medicina58030369

**Published:** 2022-03-02

**Authors:** Hyungdon Lee, Jae Min Lim, Jongwook Lee, Soo-Ki Kim, Taehun Lee

**Affiliations:** 1 Department of Internal Medicine, Chuncheon Sacred Heart Hospital, Hallym University College of Medicine, Sakju-ro 77, Chuncheon-si 24253, Gangwon-do, Korea; easydr@hanmail.net; 2 Department of Nursing Science, Hallym Polytechnic University, Janghak-gil 48, Dong-myeon, Chuncheon-si 242120, Gangwon-do, Korea; woalsdl0308@naver.com; 3 Department of Laboratory Medicine, Konyang University Hospital, Gwanjeodon-ro 158, Seo-gu, Daejeon-si 35365, Korea; lee423619@hanmail.net; 4 Department of Microbiology, Wonju College of Medicine, Yonsei University, Ilsan-ro 20, Wonju-si 26426, Gangwon-do, Korea; 5 Department of Emergency Medicine, Chuncheon Sacred Heart Hospital, Hallym University College of Medicine, Sakju-ro 77, Chuncheon-si 24253, Gangwon-do, Korea

**Keywords:** delta neutrophil index, sepsis, myeloperoxidase, cecal ligation and puncture

## Abstract

Sepsis is an emergent infectious disease and a leading cause of death despite immediate intervention. While Delta neutrophil index (DNI) and myeloperoxidase (MPO) are known as a prodiagnostic marker of sepsis, the preclinical evidence of the best marker of sepsis is unclear. For this, using a well-designed cecal ligation and puncture (CLP)-induced sepsis mouse model, we comparatively measured the level and cost-effectiveness of sepsis biomarkers such as DNI, myeloperoxidase (MPO), procalcitonin (PCT), and tumor necrosis factor-alpha (TNF-α). First, we found that the optimal time point for early detection is at 6 h, 24 h post-CLP. Strikingly, the peak level and fold change of DNI was revealed at 24 h, further showing the best fold change as compared with other biomarker levels. Given the fold change at 6, 24 h, PCT was next to DNI. Third, a cost-effectiveness survey showed that DNI was the best, with PCT next. Further, DNI level was moderate positively associated with PCT (ρ = 0.697, *p* = 0.012) and TNF-α (ρ = 0.599, *p* = 0.040). Collectively, these data indicate that DNI in CLP-induced sepsis mice is as effective as the existent inflammatory biomarkers such as MPO, PCT and TNF-α to predict the prognosis of sepsis. This might have clinically important implications that DNI is cost effective, thus quickly and rationally applying to diverse types of imminent sepsis regardless of species. This might be the first report on the validity of DNI in preclinical CLP-induced murine sepsis.

## 1. Introduction

Sepsis is an emergent infectious disease and a leading cause of death despite immediate intervention [1]. Thus, early differentiation of sepsis, culture of the pathogen of the sepsis, and treatment with broad-spectrum antibiotics are the critical points of life-saving care for sepsis patients [2,3]. Of these points, early differentiation is more important than other two points because not all of the sepsis-suspected patient are truly septic [4]. For a prompt theranostic intervention, traditionally the clinical scoring system and the usage of biomarkers have been adopted. Compared to the scoring system, the biomarker rating has definite advantages: a simple process/procedure and quality of prediction. Aside from this, since the drastic surge of sepsis due to global outbreaks of severe acute respiratory failure syndrome-associated coronavirus 2 (SARS-CoV-2), consequently the demand or necessity has been increased for better surrogate prodiagnostic markers than the existent sepsis biomarkers such as myeloperoxidase (MPO), procalcitonin (PCT), and tumor necrosis factor-alpha (TNF-α), which is a soluble molecule, a so-called non-cellular (molecular) index [5]. While those three biomarkers have, respectively, a different molecular nature such as enzymes, hormones, and cytokines, they have common biological features: a significant increase in level in sepsis [6,7,8]. Thus, they have been widely used as a prodiagnostic marker of sepsis. On the contrary to these molecular indexes, cellular indexes such as the delta neutrophil index (DNI) and immature granulocytes (IG) percentage (%) are not molecular, but cellular fractionation indexes [9]. As this cellular index is easily, quickly, accurately, and reproducibly detectable through an automatic analyzer, its application and demand has been expanded in urgent sepsis-related milieu. Given this, we hypothesized that cellular biomarkers would be superior or equivalent to non-cellular ones from the perspective of predictability and cost-effectiveness in sepsis prodiagnosis.

Of the cellular biomarkers, delta neutrophil index (DNI) is well documented as a beneficial marker in determining the occurrence of candidemia [10] with systemic inflammatory response syndrome (SIRS) and a marker of disease severity in sepsis patients [11,12,13]. DNI via an automatic hemanalyzer is easily calculated by the cellular formula of delta neutrophil index = (neutrophil [%] + eosinophil [%] − polymorphonuclear neutrophil [PMN][%]). Moreover, the time taken to measure DNI index is only 1 min. Despite these benefits, there have since been a few preclinical horse [14] and dog studies [15] to justify DNI application to human sepsis, and still there is a limitation in using DNI as a universal prodiagnostic marker of human sepsis because of weak evidence. To address this, we comparatively measured the level and cost-effectiveness of sepsis biomarkers such as DNI, MPO, PCT, and, TNF-α in a CLP-induced sepsis mouse model mimicking human sepsis.

## 2. Material and Methods

### 2.1. Animals

Male ICR mice were obtained from KOATECH (KOATECH, Pyeongtaek, Korea). We used males aged 7–8 weeks, weighing 25–30 g. The animal experiment was performed according to the national ethical guidelines and was approved by the Institutional Animal Care and Use Committee of Hallym university (IACUC approval number; HallymR1 (2021-31)).

### 2.2. Materials

MPO and TNF-α ELISA assay kits were purchased from Thermo Fisher Scientific Inc. (Waltham, MA, USA). PCT ELISA assay kits were purchased from MyBiosource, Inc. (San Diego, CA, USA).

### 2.3. Induction of Sepsis

Sepsis was induced following our previously published modification of CLP [16]. First, ICR mice were anesthetized with intraperitoneal injection of tribromoethanol (50 μg/kg). After full anesthesia, the lower abdomen was shaved using an electric shaver and disinfected with betadine solution then a 70% alcohol swab. In the longitudinal direction, a midline incision was made using a scalpel and the incision was extended into the peritoneal cavity using scissors. After intramuscular, fascial, and peritoneal incision, the cecum was located and exposed outside. Cecum was ligated with 6.0 silk sutures at the base below the ileo-cecal valve and was perforated once or twice with a 20-G needle. Then, the cecum was squeezed and a small amount of feces extruded. After that, the cecum was returned to the abdomen and peritoneum and the fascia and skin were closed via simple running sutures. Immediately post-CLP, 1ml of 0.9% saline was administered subcutaneously for fluid resuscitation.

We provided food and water placed on the bottom of cage. Mice were monitored every 6 h for survival for one to two days or euthanized at different time points for analysis of different parameters.

### 2.4. Whole Blood DNI, Serum MPO, PCT, and TNF-α Assay

We checked the baseline DNI level of mice by sham operation to rule out the increase in CRP associated with the inflammation effect of CLP surgery itself. The total number of ICR mice examined for sham surgery was 40. The blood samples were collected by retrobulbar plexus puncture of mice under general anesthesia (intraperitoneal tribromoethanol injection (50 μg/kg)). For DNI measurement, whole blood collected at each time period before and 6 h, 24 h, and 48 h after CLP was placed in the EDTA tube and delivered by icebox to NEODIN co. Ltd. to check the DNI. The DNI was measured by an automatic cell analyzer (ADVIA 2120 Hematology System, Siemens Healthcare Diagnostics, Forchheim, Germany). The calculation method of DNI used two independent white blood cell (WBC) analysis methods: MPO channel and lobularity/nuclear density channel. The formula for calculating DNI is neutrophil and eosinophil subfraction in MPO channel–polymorphonuclear neutrophil (PMN) subfraction in the nuclear lobularity channel [17]. For serum MPO, PCT, and TNF-α measurement, whole blood collected with the same protocol as DNI measurement was centrifuged at 5000 RPM for 15 min to obtain serum. Thereafter, MPO, PCT, and TNF-α levels were measured with commercial ELISA assay kits.

### 2.5. Measurement of Cost-Effectiveness

To find out the cost of sepsis biomarkers, we contacted the device manager of the institute, or the sales manager in case of commercial kits. To measure the test time of sepsis biomarkers, we directly measured or observed the test time of each biomarker.

### 2.6. Statistical Analysis

Continuous variables are presented as the mean ± standard deviation (SD) and compared using ANOVA test. Categorical variables are presented as percentages and compared using chi-square or Fisher’s exact test. Spearman’s rank correlation coefficient was used to evaluate the association between DNI, MPO, PCT, and TNF-α. A *p*-value of less than 0.05 was decided as statistically significant. The Kaplan–Meier method with log-rank test was used to compare between-group differences in mortality rates. All statistical analyses were performed using IBM SPSS statistics for Windows, version 23.0 (IBM Corp., Armonk, NY, USA).

## 3. Results

**Figure 1:**
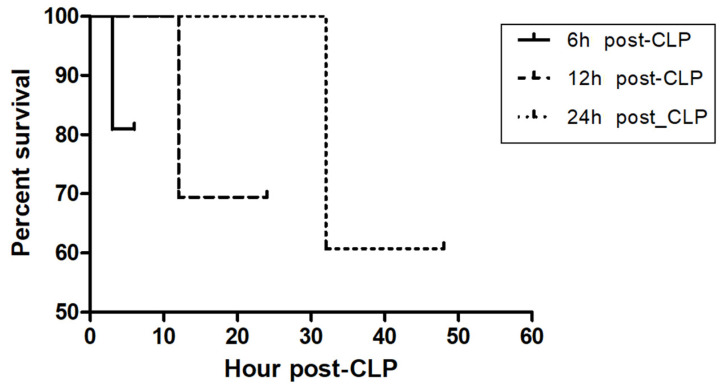
Kaplan–Meier curve in CLP-induced sepsis mice following different CLP timing. To confirm the validity of our CLP-induced sepsis as well as the optimal time to predict the severity of sepsis by measuring biomarkers level, we examined the lethality [[the dead number (percentage: %)]] of CLP-induced septic mouse at 6 h, 24 h, and 48 h post-CLP. It was 4 (19%) out of 21, 11 (30.5%) out of 36, and 11 (39.2%) out of 28 (Table 1). Log-rank test between each group was significant (*p* = 0.0018).

The total number of mice for study was 165. The number of mice to calculate the average of the baseline CLP level of mice by sham operation was 40. The baseline DNI level of mice by sham operation was 1.5 ± 1.1%. To confirm the validity of our CLP-induced sepsis as well as the optimal time to predict the severity of sepsis by measuring biomarker levels, we examined the lethality (the dead number (percentage: %)) of CLP-induced septic mice at 6 h, 24 h, and 48 h post-CLP. It was 4 (19%) out of 21, 11 (30.5%) out of 36, and 11 (39.2%) out of 28 (Table 1). Further, the Kaplan–Meier method with log-rank test clearly proved the validity of our CLP-induced sepsis mouse model (*p* = 0.0018, Figure 1). From the perspective of the representative time for the severity of sepsis, we speculate that a time point corresponding to 30% or less lethality for early detection is optimal to compare biomarker levels, thus proven at 6 h, 24 h post-CLP. Next, we measured the level and fold change of sepsis biomarkers such as DNI, MPO, PCT, and TNF-α at baseline, 6 h, 24 h and 48 h post-CLP. The baseline level of all biomarkers was less than 0.5 (% or ng.ml), and stable with less variation. In all biomarkers, the difference between the duration of post-CLP was significant as *p* < 0.02 (Table 2). The pattern of time kinetics in each marker was the same except DNI: a time-dependent increase in MPO, PCT, and TNF-α, albeit time-independency in DNI (Table 2). Strikingly, the peak level and fold change of DNI was revealed at 24 h, further showing the best fold change as compared with other biomarker levels. Given the fold change at 6 and 24 h, PCT was next to DNI (Table 2). Third, to check the clinical applicability, we compared the cost-effectiveness between sepsis biomarkers by way of surveying cost and test time. Table 3 showed that in cost-effectiveness, DNI was the best, and next was PCT. Finally, Table 4 shows that using Spearman’s rank correlation, the level of DNI was moderate positively correlated with that of PCT (*ρ* = 0.697, *p* = 0.012) and TNF-α (*ρ* = 0.599, *p* = 0.040).

## 4. Discussion

These data indicate that DNI in CLP-induced sepsis mice is as effective as the existent inflammatory biomarkers such as MPO, PCT and TNF-α to predict the prognosis of sepsis. Prior to the clinical application of DNI, clinical scoring systems such as APACH score, SOFA score and SAPS score have been widely used [18,19]. However, such scoring systems need many parameters such as blood pH, Glasgow coma scale and vital signs to have a final score and take time to draw arterial and venous blood and test electrolytes [20]. Consequently, those complex processes may be at risk of some complications, such as subcutaneous hematoma and pseudo-aneurysm after blood sampling and time spent of at least 6 h with higher cost. In addition, the greatest weakness is that from this scoring system, the prediction of sepsis and its prognosis is not feasible [21]. To compensate for this, molecular biomarkers as a non-cellular source have been introduced as early prodiagnostic markers for human sepsis, which have overcome the drawbacks of the scoring systems [22]. However, there is room for the well-designed evidence and cost-effectiveness to justify universal usage in human sepsis. Despite only intermittent human study, there have been canine and equine sepsis reports. Unfortunately, these are not well designed in terms of natural sepsis, like in human cases. Thus, to minimize the error or heterogeneity out of the inherent immune response of sepsis, we devised an artificially planned murine sepsis model using CLP in ICR mice, which has been already proven to be consistent, reproducible, and rational in the context of simulating human sepsis. First, a representative time for the severity of sepsis was proven at 6 h and 24 h post-CLP. This is supported by a previous human study [23]. Since the test time of our molecular biomarkers is maximally 6 h, 6 h of bleeding is the only choice for the earliest time point. In other 48 h post-CLP sepsis mouse models, there were several reports on maximally 80% or higher mortality; in fact, we speculate that at that late, imminent time point, bleeding to predict the severity of sepsis is not reasonable from the point of degrading prodiagnostic value.

Next, time kinetics of comparative levels between biomarkers showed that the pattern of DNI (24 h peak, time independency) is differential from that of MPO, PCT, and TNF-α (48 h peak, time dependency). Strikingly, the peak level and fold change of DNI was revealed at 24 h, further showing the best fold change as compared with other biomarker levels (Table 2). Given the fold change of all biomarkers at 6 and 24 h, DNI was best (30.1, 159.3), and PCT was next (20, 62). By contrast, the fold change of MPO and TNF-α at 6 and 24 h was quite lower than that of DNI and PCT. Considering the time kinetics from baseline to 24 h post-CLP, the rank based on the incremental velocity of biomarker level is DNI, PCT, MPO, and TNF-α in descending order. When examining the comparative profiling between sepsis-related biomarkers, these data might imply that the optimal time point to measure biomarkers as well as the velocity or fold change of biomarker level with time kinetics would be essential to select the feasible sepsis biomarker as well as to predict the severity and progress of sepsis by the given biomarker.

Third, the cost-effectiveness between sepsis biomarkers indicated that the rank of clinically applicable biomarkers is DNI, PCT, TNF-α, and MPO in descending order (Table 3). Further, the positive correlation with the level of PCT and TNF-α by that of DNI might importantly suggest the potential usage of surrogate cellular biomarkers.

In addition, synthesizing our data and speculation, DNI as a cellular index has incomparable advantages over non-cellular indexes or biomarkers: it is cost-effective, simple, accurate, reproducible, and easily applicable to diverse type of sepsis or infection irrespective of species. Furthermore, our statement could be justified by the rational minimization of data error via a well-designed CLP-sepsis murine model, which is evidenced by log-rank test (Figure 1) and ANOVA (Table 2). However, our study has a clear limitation. As the CLP procedure needs much skill and expertise, the mortality rate might vary depending on the protocol and operator. Then, the optimal time point for bleeding will vary. If more bleeding before mouse death is possible, a better time kinetic of biomarker level will be warranted.

## 5. Conclusions

Collectively, these data indicate that DNI in CLP-induced sepsis mice is as effective as the existent inflammatory biomarkers such as MPO, PCT and TNF-α to predict the prognosis of sepsis. This might have clinically important implications that DNI is cost-effective, thus quickly and rationally applying to diverse types of imminent sepsis regardless of species. This might be the first report on the validity of DNI in preclinical CLP-induced murine sepsis.

## Figures and Tables

**Table 1:** Lethality of CLP-induced sepsis mice.

	Dead Mice (Number) (%)	Live Mice (Number) (%)	Total Mice (Total Number) (%)
6 h post-CLP	4 (19)	17 (81)	21 (100)
24 h post-CLP	11 (30.5)	25 (69.5)	36 (100)
48 h post-CLP	11 (39.2)	17 (60.8)	28 (100)

**Table 2:** Comparative level of DNI, MPO, PCT, and TNF-α in sepsis mice following the duration of post-CLP.

DNI (%) MPO (ng/mL) PCT (ng/mL) TNF-α (ng/mL)				
(Fold Increased: Given Hour/Baseline)				
Baseline	0.4 ± 0.7 (1)	0.2 ± 0.1 (1)	0.4 ± 0.2 (1)	0.15 ± 0.15 (1)
6 h post-CLP	12.3 ± 5.6 (30.1)	0.13 ± 0.2 (0.7)	8.0 ± 5.4 (20)	0.7 ± 0.3 (4.7)
24 h post-CLP	63.7 ± 7.2 (159.3)	2.3 ± 0.4 (11.5)	24.7 ± 5.3 (62)	1.0 ± 0.5 (6.7)
48 h post-CLP	31.4 ± 19.9 (78.5)	29.3 ± 1.1 (146.5)	84.1 ± 12.1 (210.3)	34.4 ± 4.5 (229.3)
*p* value	0.021	0.023	0.024	0.016

Delta neutrophil index (DNI); myeloperoxidase (MPO); procalcitonin (PCT); tumor necrosis factor-alfa (TNF-α). The sample number is the same as the number of live mice group except baseline group (N = 40). Difference between the duration of post-CLP was significant as *p* < 0.02 in all biomarker group.

**Table 3:** Cost-effectiveness between sepsis biomarkers.

	DNI	PCT	TNF-α	MPO
Cost (US Doller/)/Test	8.5	26.66	49.1	51.315
Test time (Hour)	0.02	3	6	6

Delta neutrophil index (DNI); myeloperoxidase (MPO); tumor necrosis factor-alfa (TNF-α); procalcitonin (PCT).

**Table 4:** Spearman’s rank correlation between sepsis biomarkers such as DNI, MPO, TNF-α, and PCT.

	DNI	MPO	TNF-α	PCT
Spearman’s rho DNI rho	1.000	0.442	0.599 *	0.697 *
*p* value (two tailed)		0.150	0.040	0.012
Number	12	12	12	12
MPO rho	0.442	1.000	0.743 **	0.809 **
*p* value (two tailed)	0.150		0.006	0.001
Number	12	12	12	12
TNF-α rho *p* value (two tailed) Number	0.599 * 0.040 12	0.743 ** 0.006 12	1.000 12	0.923 ** 0.000 12
PCT rho *p* value (two tailed) Number	0.697 * 0.012 12	0.809 ** 0.001 12	0.923 ** 0.000 12	1.000 12

Delta neutrophil index (DNI); myeloperoxidase (MPO); tumor necrosis factor-alfa (TNF-α); procalcitonin (PCT). * Spearman’s rank correlation is significant in *p* < 0.05 (Two tailed). ** Spearman’s rank correlation is significant in *p* < 0.01 (Two tailed).
